# Research trends of nanotechnology in coronary heart disease: a bibliometric analysis from 2012 to 2025

**DOI:** 10.3389/fphar.2026.1931509

**Published:** 2026-09-30

**Authors:** Hui-Fang Kuang, Song-Yan Tie, Qi-Zhi He, Jie Li, Ling-Li Chen

**Affiliations:** 1 School of Integrative Traditional Chinese and Western Medicine, Hunan University of Chinese Medicine, Changsha, China; 2 School of Basic Medical Science and Hunan Provincial Key Laboratory of the Research and Development of Novel Pharmaceutical Preparations and Hunan Provincial University Key Laboratory of the Fundamental and Clinical Research on Functional Nucleic Acid, Changsha Medical University, Changsha, China; 3 Hunan University of Chinese Medicine Library, Hunan University of Chinese Medicine, Changsha, China; 4 Hunan Provincial Key Laboratory of Vascular Biology and Translational Medicine and Medical School of Hunan University of Chinese Medicine, Changsha, China

**Keywords:** bibliometric, coronary heart disease, immunity, inflammation, macrophage, nanomedicine

## Abstract

**Background:**

Coronary heart disease (CHD) remains a leading cause of mortality worldwide, with conventional therapeutic strategies limited by non-specific targeting, low bioavailability, and systemic off-target effects. Nanomaterials, leveraging their unique physicochemical properties, have shown immense potential in CHD diagnosis and therapy. However, the global research landscape, knowledge structure, and thematic evolution of nanomaterials in CHD have not been systematically evaluated.

**Methods:**

Relevant publications from 2012 to 2025 were retrieved from the Web of Science Core Collection and Scopus databases. Bibliometric and visualization analyses were performed using CiteSpace, VOSviewer, and Scimago Graphica. The study evaluated publication trends, international and institutional collaboration networks, co-occurrence patterns, citation characteristics, and emerging research themes through keyword clustering and burst detection.

**Results:**

A total of 2,709 publications were included, demonstrating a continuous increase in research activity during the study period. China and the United States were the leading contributors in publication output and international collaboration. Major institutional contributors included Harvard University, Massachusetts General Hospital, and the Chinese Academy of Sciences. Keyword co-occurrence and burst analyses identified several major research directions, including nanobiosensing, molecular imaging, targeted nanoparticle delivery, and regenerative medicine. Among these evolving themes, immune-inflammatory mechanisms, macrophage/monocyte regulation, oxidative stress, and microenvironmental modulation have emerged as increasingly prominent research directions in nanotechnology-based CHD investigation.

**Conclusion:**

This bibliometric analysis provides an overview of the global development and knowledge evolution of nanotechnology research in CHD from 2012 to 2025. The findings indicate that immune-inflammatory mechanisms represent an emerging research theme within the broader landscape of cardiovascular nanotechnology. The growing attention toward immune modulation and disease-specific microenvironment regulation identified in this study may represent important directions for future translational research in cardiovascular nanotechnology.

## Graphical Abstract

**Figure:**
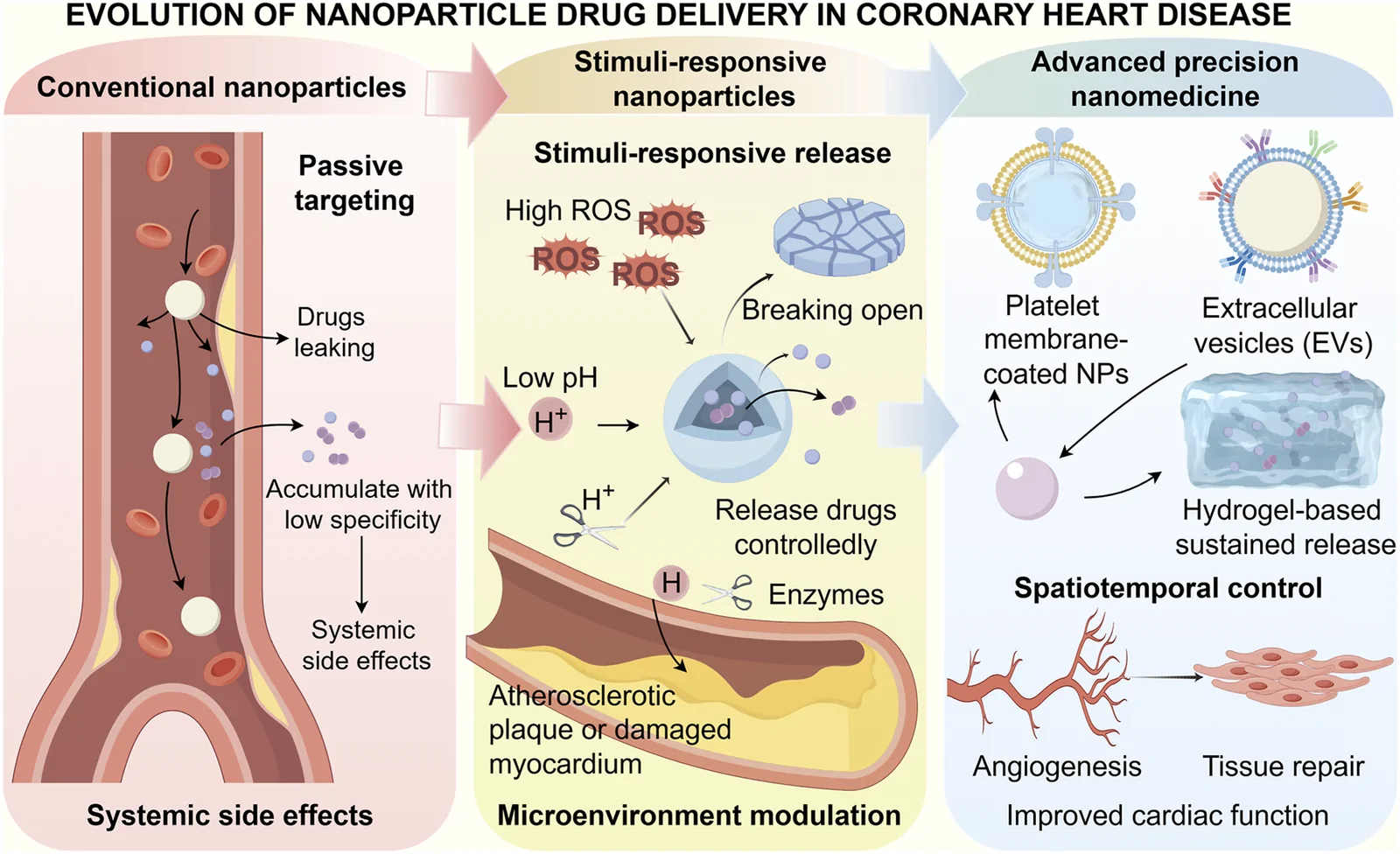
(By Figdraw 2.0). Note: Conceptual summary of research evolution identified by bibliometric analysis.

## 1 Introduction

Cardiovascular diseases (CVDs) remain one of the leading causes of mortality worldwide. Among them, coronary heart disease (CHD), primarily driven by atherosclerosis (AS), poses a substantial threat to global health and quality of life and represents a major public health burden (Zhu et al., 2021). The pathophysiology of AS is characterized by the subendothelial accumulation of low-density lipoprotein, the formation of macrophage-derived foam cells, and the progressive development of vulnerable plaques (Psarros et al., 2012). A major clinical challenge lies in the difficulty of detecting early-stage, subclinical plaques, which are often only diagnosed following acute cardiovascular events (Sebatana et al., 2025). Although conventional therapeutic strategies—including statins, antithrombotic therapy, and invasive revascularization—substantially reduce cardiovascular event rates and achieve acute reperfusion, their capability to directly induce atherosclerotic plaque regression or promote long-term myocardial regeneration remains constrained by systemic clearance and limited local retention (Peters et al., 2021). Therefore, the development of innovative preventive and diagnostic strategies with enhanced specificity, sensitivity, and safety is urgently needed.

Nanotechnology offers promising solutions to overcome the limitations of traditional cardiovascular therapeutics (Cheng et al., 2023). Owing to their large specific surface area, tunable physicochemical properties, and versatile surface functionalization capabilities, nanomaterials have been extensively applied in molecular imaging, targeted drug delivery, and biosensing (Amin et al., 2015; Pretorius et al., 2021; Diez-Pascual and Rahdar, 2022). For example, ultrasmall superparamagnetic iron oxide nanoparticles, used as contrast agents in magnetic resonance imaging (MRI), can passively accumulate in atherosclerotic plaques via the enhanced permeability and retention effect, thereby improving the detection of vulnerable lesions (Juenet et al., 2015). Furthermore, through advanced surface engineering strategies—such as ligand–receptor-mediated targeting and biomimetic membrane coating—nanocarriers can actively deliver therapeutic agents to activated endothelial cells, foam cells, and collagen-rich lesion sites, enabling spatially controlled drug release (Song et al., 2019; Zia et al., 2020).

Beyond inhibiting plaque progression, nanotechnology also demonstrates significant potential in cardiac repair and regenerative medicine following myocardial infarction (MI). In particular, extracellular vesicles (EVs), as naturally derived, cell-free nanocarriers, have emerged as an effective approach in cardiac tissue engineering to promote endogenous myocardial regeneration and therapeutic angiogenesis (Arifin et al., 2022; Zhang et al., 2022). As carriers of bioactive molecules—including proteins, lipids, and nucleic acids—EVs can traverse biological barriers and, when combined with advanced bioengineering strategies, exhibit enhanced *in vivo* stability and targeting specificity (Briffault et al., 2024).

Bibliometric analysis, through the systematic evaluation of large-scale scientific literature, provides a comprehensive and objective perspective on research development. This approach facilitates a deeper understanding of the evolution and current landscape of nanomaterial applications in CHD and supports the identification of emerging trends and future research directions (Cao et al., 2024). Over the past decade, the application of nanomaterials in CHD research has increased substantially. Therefore, this study aims to systematically investigate research trends, the evolution of targeting strategies, and the key challenges associated with the clinical translation of nanomaterials in CHD from 2012 to 2025 using bibliometric and visualization analyses, thereby providing a macroscopic scientific framework and reference for future investigations.

## 2 Materials and methods

### 2.1 Data sources and search strategy

To ensure the comprehensiveness and rigor of the bibliometric analysis, this study adopted an analytical method integrating two databases (Hu et al., 2026). This study was designed as a bibliometric and scientometric analysis to characterize the global research landscape, knowledge structure, collaboration patterns, and emerging research trends in nanotechnology related to coronary heart disease. Unlike conventional systematic reviews or meta-analyses, this study did not aim to evaluate clinical effectiveness, perform risk-of-bias assessments, or conduct qualitative appraisal of individual studies. The Web of Science Core Collection (WoSCC) is widely recognized for its comprehensive multidisciplinary coverage and high-quality citation indexing. Scopus, one of the largest interdisciplinary abstract and citation databases worldwide, provides a large volume of high-quality bibliographic data (Baas et al., 2020). By integrating multiple databases for bibliometric analysis, this study aimed to construct a more comprehensive academic landscape of the field and present the research results in a multidimensional and more accurate manner. This approach helps overcome the inherent inclusion bias and data limitations of a single database, thereby significantly improving the reliability of the analysis results (Lin et al., 2019). The search formula of the WoSCC is: TS =(“nanoparticle” OR “nanomaterial” OR “nanocarrier” OR “nanomedicine” OR “nanosystem” OR “nanostructure” OR “nanocrystal” OR “nanoformulation”) AND (“coronary heart disease” OR “coronary artery disease” OR “ischemic heart disease” OR “myocardial infarction” OR “acute coronary syndrome” OR “coronary disease” OR “CHD” OR “CAD”). The search formula in the Scopus database is: TITLE-ABS-KEY ((“nanoparticle” OR “nanomaterial” OR “nanocarrier” OR “nanomedicine” OR “nanosystem” OR “nanostructure” OR “nanocrystal” OR “nanoformulation”) AND (“coronary heart disease” OR “coronary artery disease” OR “ischemic heart disease” OR “myocardial infarction” OR “acute coronary syndrome” OR “coronary disease” OR “CHD” OR “CAD”)). The search was restricted to published English-language articles from 1 January 2012, to 31 December 2025, published only in English. The retrieved literature was exported in “full records and cited literature” format. Two independent researchers screened the retrieved records by reviewing titles and abstracts, excluding irrelevant and duplicate studies. The literature identification and screening process is presented using a PRISMA-style flow diagram (Figure 1) to transparently describe the retrieval, screening, and inclusion procedures. However, this diagram was used solely for reporting transparency and does not represent a formal PRISMA/Cochrane systematic review or evidence synthesis framework. During this process, 338 records were excluded, leaving 2,709 publications for subsequent analysis (Figure 1). Any disagreements during the screening process were resolved by the corresponding author (L.L.C.). The procedures for merging and data cleaning of records from the two databases followed a standardized bibliographic workflow (Kuang et al., 2025). Eligible records retrieved from WoSCC and Scopus were exported in compatible formats (WoSCC: plain text format; Scopus: plain text and RIS formats) and subsequently imported into EndNote for reference management and duplicate identification. EndNote was used to identify potential duplicate records based on multiple bibliographic fields, including title, authors, publication year, journal information, volume/pages, and DOI when available. Because differences in metadata formatting may occur between databases, all automatically identified duplicate records were manually reviewed and verified to confirm true duplicates before removal. After the deduplication process, 75 duplicate records were removed, resulting in 2,709 unique publications for subsequent bibliometric analyses. The final dataset consisted of 931 records from WoSCC and 1,778 records from Scopus. Citation information and cited references from the retained records were preserved for subsequent citation analysis and network visualization. Studies were limited to English-language publications indexed in WoSCC and Scopus.

**FIGURE 1:**
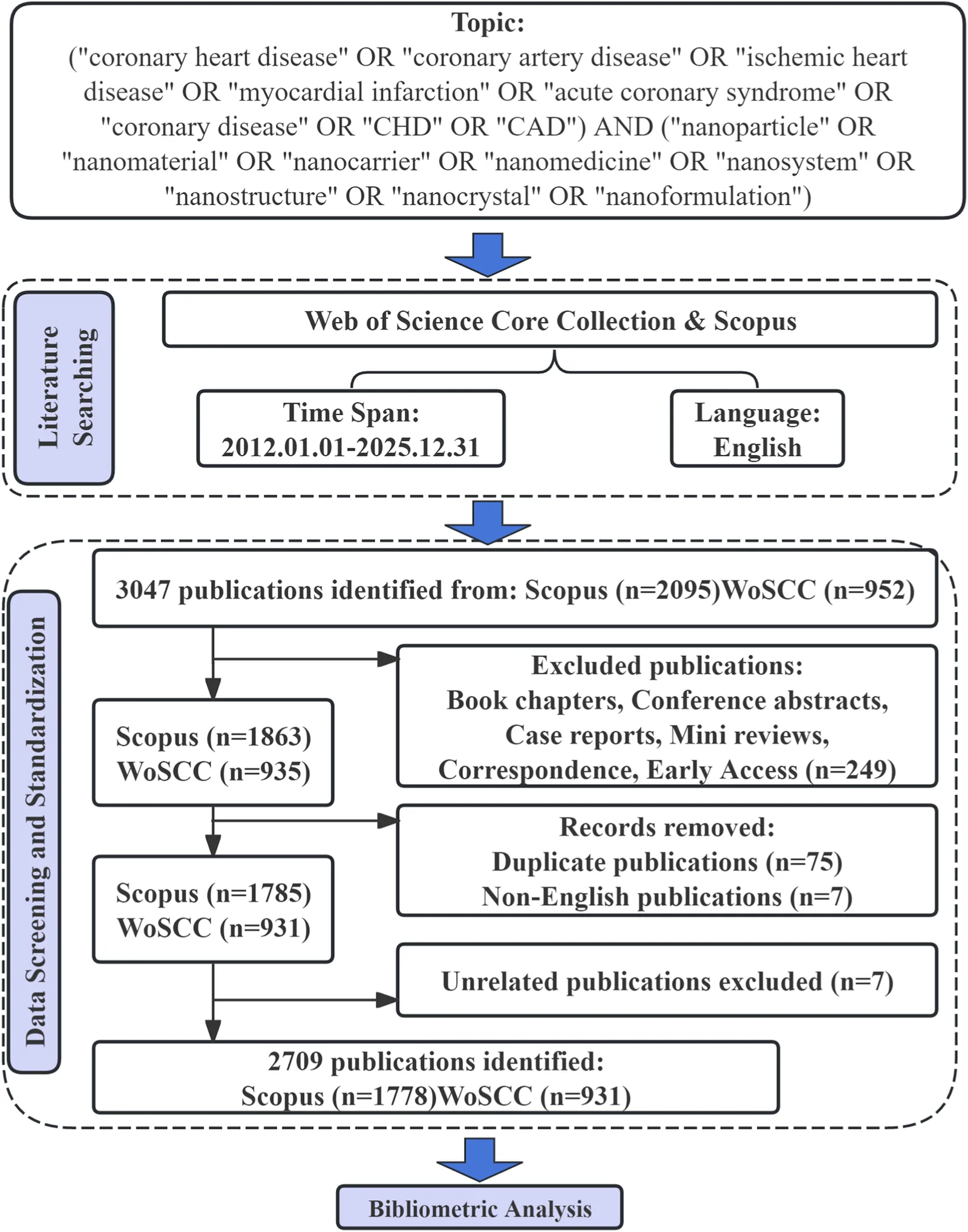
Flowchart of literature selection based on the PRISMA guidelines.

### 2.2 Data analysis and visualization

This study employed Microsoft Excel 2021, VOSviewer (version 1.6.18), CiteSpace (version 6.4.R2 Advanced), and Scimago Graphica (beta 1.0.52) as the bibliometric analysis tools. Before network construction, the final dataset was carefully reviewed and standardized to improve data consistency and analytical accuracy. Bibliographic information, including author names, institutional affiliations, countries/regions, journal information, and keywords, was checked and standardized where necessary. Synonymous terms, abbreviations, and singular/plural variants of keywords were merged when appropriate, and redundant or irrelevant terms were removed during keyword curation. Cited references were also reviewed to minimize inconsistencies caused by variations in bibliographic records from different databases. CiteSpace (version 6.4.R2 Advanced) was used for bibliographic data conversion, network construction, and visualization of the intellectual structure, temporal evolution, and emerging research trends of nanotechnology in coronary heart disease. The analysis period was set from January 2012 to December 2025, with 1 year per time slice. The selection criterion was set using the Top N method (Top 50), and the node types included countries, institutions, authors, cited authors, cited references, journals, and keywords. Co-occurrence, collaboration, and co-citation networks were generated to identify major contributors, knowledge structures, and research hotspots. For network simplification, pruning was performed using Pathfinder and Pruning Sliced Networks, while other parameters were maintained as default settings. Keyword clustering was conducted using the log-likelihood ratio (LLR) algorithm, and timeline visualization was generated to illustrate the evolution of research themes. Keyword burst detection was performed to identify emerging research topics with rapidly increasing attention (Chen, 2006). In CiteSpace analysis, betweenness centrality was used to evaluate the bridging role of nodes within the network, with higher centrality values indicating a more prominent position in connecting different clusters. The reliability and significance of clustering structures were assessed using modularity Q value and weighted mean silhouette coefficient, where Q > 0.3 and silhouette >0.5 indicate acceptable clustering quality.(Hou et al., 2025).

VOSviewer was used to construct and visualize bibliometric networks, including country, institutional, journal, author, and keyword co-occurrence networks, as well as co-citation networks (van Eck and Waltman, 2010). For collaboration analyses of countries, institutions, and journals, the minimum number of documents was set to 5. For co-citation analyses of authors, journals, and references, the minimum number of citations was set to 20. Other network construction settings were maintained according to the default parameters of VOSviewer unless otherwise specified.

Journal impact factor (IF) data were obtained from the 2025 Journal Citation Reports (JCR). Microsoft Excel 2021 was used to generate annual publication trends, citation-related analyses, and publication trends of the top ten productive countries. Origin 2025b was used to generate heat maps illustrating annual publication outputs of the top ten countries. Scimago Graphica was used for selected collaboration and geographical visualization. Through these analyses, this study constructed a multidimensional knowledge network, characterized the developmental trajectory of nanotechnology research in coronary heart disease, and identified major research themes and emerging hotspots.

## 3 Result

### 3.1 Literature search results

Our search retrieved 3,047 records, with 2,709 articles and reviews meeting the inclusion criteria after screening.

### 3.2 Analysis of publications and citations

The number of publications is a key indicator for evaluating research progress and the development of a scientific field. The temporal distribution of annual publications related to nanomaterials and coronary heart disease is illustrated (Figure 2). Overall, the annual publication output demonstrates a fluctuating yet upward trend. Notably, publications from the past 5 years account for 62.13% of the total output. Globally, the number of annual publications increased from 52 in 2012 to 432 in 2025, corresponding to an average annual growth rate of 17.63%, indicating an overall acceleration in research productivity. This recent upward trajectory reflects a growing academic interest and suggests that the field is gradually reaching a stage of maturation.

**FIGURE 2:**
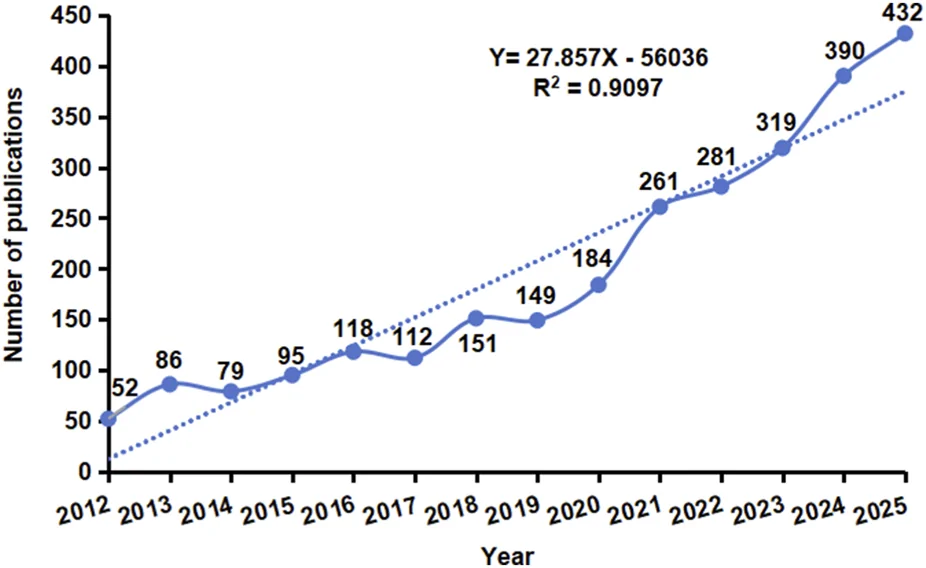
Annual publications on nanomaterials and CHD from 2012 to 2025. A fitted polynomial curve (R^2^ = 0.9097) demonstrates the overall increasing trend in research output. The R^2^ value indicates a strong goodness of fit, supporting the reliability of the observed trend. This curve is presented for descriptive visualization purposes rather than for prediction.

### 3.3 Analysis of countries/regions

Figures 3A,B illustrate the annual publication trends of the top 10 most productive countries. In total, 66 countries/regions have contributed to research in this field (Table 1). China ranks first in terms of total publications (n = 402, 14.84%), followed by the United States (n = 210) and India (n = 62). These countries not only demonstrate high research productivity but also occupy central positions within the global collaboration network. Although China leads in total publication output, its average citation per article is relatively low (27.02). In contrast, the United States exhibits the highest average citation per article (54.90), indicating greater academic impact. Total link strength (TLS) analysis further reveals that the United States, China, Italy, and Saudi Arabia serve as key nodes within the collaboration network. The United States has received the highest number of citations, followed by China and South Korea. Regarding international collaboration, the network visualization presented in Figure 3C provides valuable insights into global partnerships. Figure 4 further demonstrates the geographical distribution of research activity in this field, highlighting that the most prominent collaborative pattern occurs between the United States and China, which has, to some extent, facilitated international knowledge exchange.

**FIGURE 3:**
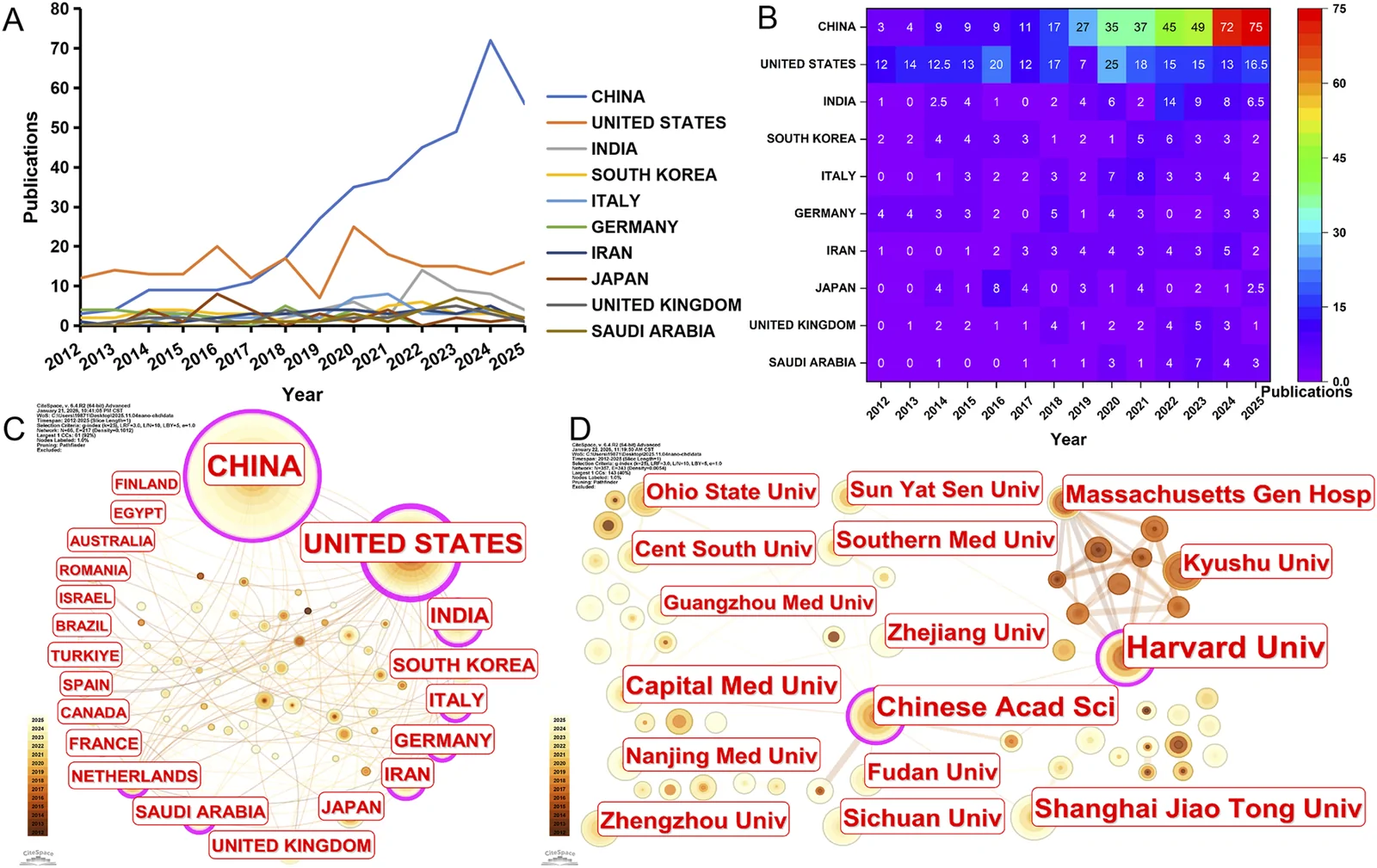
Contributions of countries and institutions to nanomaterials and CHD research. **(A)** Annual publication trends of the top 10 countries. **(B)** Heat map of national publication distribution. **(C)** International collaboration network, where node size reflects publication output and link thickness indicates collaboration strength. **(D)** Institutional collaboration network, with node size representing productivity and link thickness denoting co-authorship intensity.

**TABLE 1:** Top 10 countries/regions with the highest number of publications in the field of nanomaterials and CHD from 2012 to 2025.

Rank	Country/region	Publications	Centrality	Percentage (%)	Citation	Citation per publication	TLS	Centrality
1	CHINA	402	0.18	14.84%	10,862	27.02	92	0.18
2	UNITED STATES	210	0.58	7.75%	11,529	54.90	136	0.58
3	INDIA	62	0.2	2.29%	1,149	18.53	38	0.20
4	SOUTH KOREA	41	0.07	1.51%	2,148	52.39	21	0.07
5	ITALY	40	0.12	1.48%	2081	52.03	42	0.13
6	GERMANY	37	0.11	1.37%	1737	46.95	37	0.13
7	IRAN	36	0.13	1.33%	1,651	45.86	28	0.13
8	JAPAN	31	0	1.14%	1,515	48.87	10	0.01
9	UNITED KINGDOM	29	0.08	1.07%	959	33.07	36	0.08
10	SAUDI ARABIA	27	0.13	1.00%	1,178	43.63	40	0.13

**FIGURE 4:**
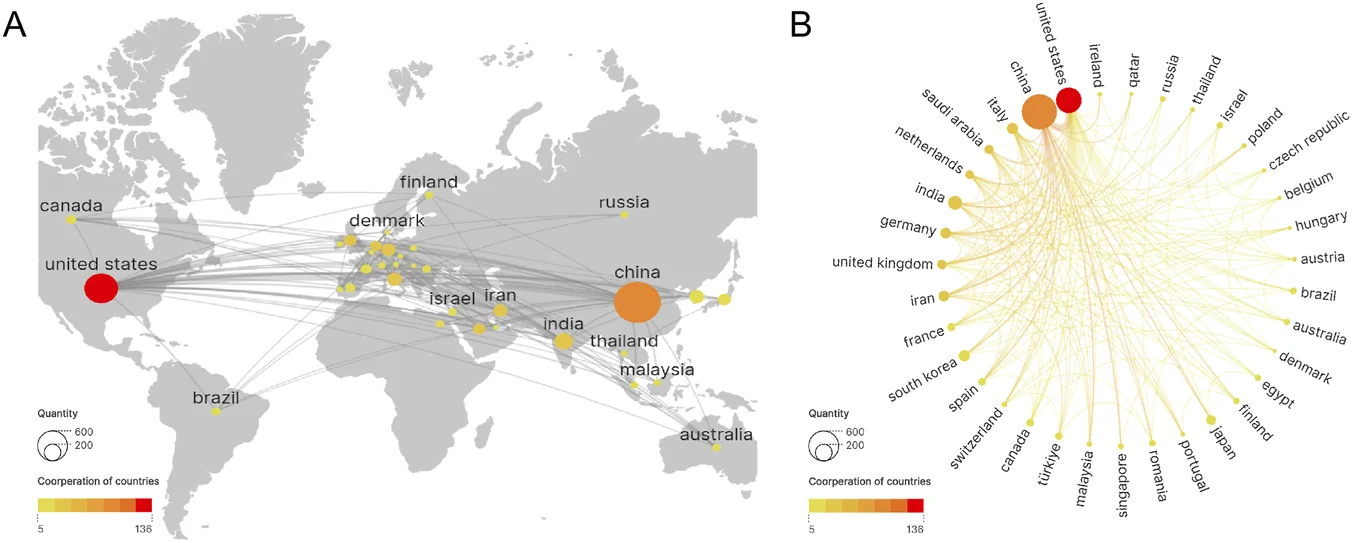
Contributions of countries on nanomaterials and CHD research. **(A,B)** Country collaboration map by Scimago Graphica.

### 3.4 Analysis of institutions

A total of 1,392 institutions have contributed publications in the field of nanomaterials and coronary heart disease, with the top 10 institutions listed in Table 2. Harvard University ranks first in terms of publication output (n = 34). VOSviewer analysis identified 76 institutions that have published five or more articles. The institutional collaboration network, visualized using CiteSpace (Figure 3D), indicates that research outputs in this field are concentrated within a relatively small number of leading institutions. Notably, a closely connected collaborative network is observed among the Chinese Academy of Sciences, Harvard University, Capital Medical University, and Shanghai Jiao Tong University. In addition, Massachusetts General Hospital and Harvard University exhibit the strongest international institutional partnership.

**TABLE 2:** Top 10 institutions of high contribution.

Rank	Institution	Publications	Centrality	Total link strength
1	Harvard University	34	0.13	108
2	Chinese Academy of Sciences	23	0.17	62
3	Shanghai Jiao Tong University	21	0.05	86
4	Capital medical University	17	0.06	86
5	Sichuan University	16	0	70
6	Massachusetts General Hospital	16	0.05	164
7	Zhengzhou University	14	0.01	81
8	Kyushu University	14	0	128
9	Southern medical University	14	0.02	39
10	Fudan University	13	0.03	85

Overall, research in this domain is predominantly conducted by research-intensive universities and globally recognized institutions with well-established medical programs and comprehensive interdisciplinary infrastructures. Leveraging their advanced research platforms and academic resources, these institutions play a central role in driving high-quality scientific output and enhancing the global impact of this field.

### 3.5 Analysis of authors

Co-cited author analysis refers to the phenomenon in which publications by two authors are cited simultaneously by a third author. A higher co-citation frequency indicates greater similarity in research focus and a closer intellectual relationship between authors (Cheng et al., 2021). Analyzing both highly productive authors and highly co-cited authors in the field of nanomaterials and coronary heart disease provides an intuitive reflection of the field’s research capacity and major hotspots. A total of 5,976 authors were involved in the included publications, among whom 38 authors published five or more articles (Figure 5). The top 10 most productive authors and the most co-cited authors are presented in Table 3. Nahrendorf, M ranked first, with 14 publications and a total of 2,220 citations. Egashira, K and Matoba, T ranked second, each with 14 publications, but with comparatively lower total citation counts (1,100 citations each). In contrast, Weissleder, R published only 7 articles but achieved a high total citation count of 1,656. Among the top three most cited productive authors, Nahrendorf, M is affiliated with Massachusetts General Hospital, while Egashira, K and Matoba, T are affiliated with Kyushu University, indicating that the latter is a key institution contributing to core research outputs in this field. However, five of the top 10 authors exhibit betweenness centrality values below 0.05, However, five of the top 10 authors exhibit betweenness centrality values below 0.05. From a network-topology perspective, low betweenness centrality indicates that these authors primarily operate as core nodes within dense, specialized local research clusters rather than serving as cross-network bridges. While this reflects a degree of thematic compartmentalization, it does not imply a total lack of collaborative effort, but rather points to opportunities for strengthening cross-disciplinary integration. As shown in Table 3, Libby, P is the most frequently co-cited author (125 co-citations), followed by Nahrendorf, M (120 co-citations), who is also the most productive author in this field. Other highly co-cited authors include Zhang, Y (120 co-citations), Wang, Y (112 co-citations), and Frangogiannis, N.G. (90 co-citations).

**FIGURE 5:**
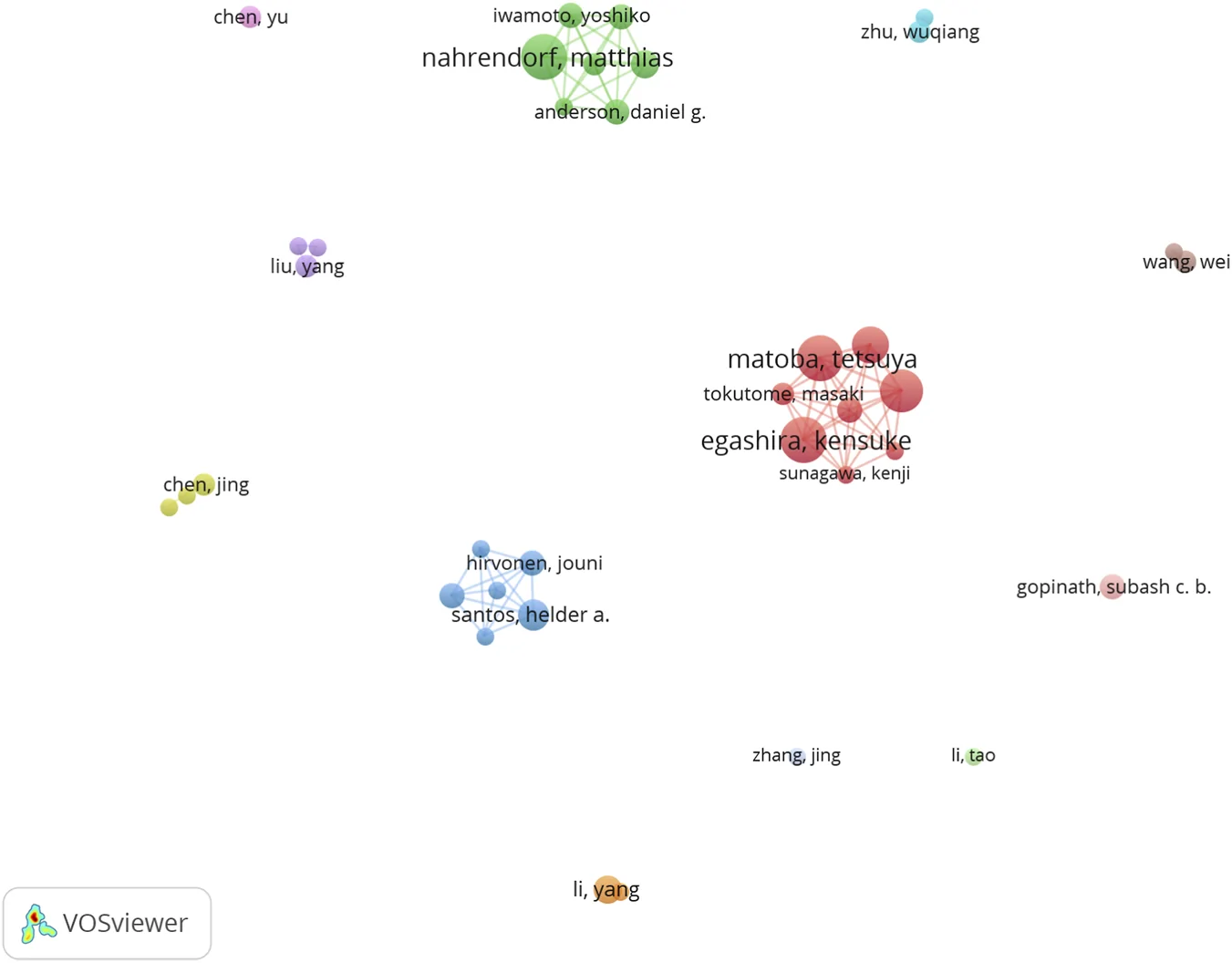
Cooperation network of authors of investigations on nanomaterials and CHD. Note: Node size represents total publication count; colors indicate distinct collaborative clusters; lines denote co-authorship ties. The network includes two primary clusters alongside minor isolated components.

**TABLE 3:** The top 10 most productive authors and co-cited authors contributed to nanomaterials and CHD.

Rank	Author	Count	Citation	TLS	Citation per publication	Co-cited author	Citation counts	Centrality
1	Nahrendorf, M	14	2,220	38	158.57	Libby, P	125	0.09
2	Egashira, K	14	1,100	60	78.57	Nahrendorf, M	120	0.1
3	Matoba, T	14	1,100	60	78.57	Zhang, Y	120	0.03
4	Nakano, K	13	1,043	59	80.23	Wang, Y	112	0.03
5	Koga, JI	11	868	48	78.91	Frangogiannis, NG	90	0.08
6	Swirski, F	8	1,178	37	147.25	Wang, J	78	0.01
7	Li,Y	8	82	1	10.25	Liu, Y	75	0.07
8	Iwamoto, Y	7	1,072	36	153.14	Wang, L	71	0.03
9	Weissleder, R	7	1,656	33	236.57	Li, Y	69	0.02
10	Anderson, D	7	1,041	28	148.71	Leuschner, F	63	0.11

Author co-occurrence network analysis aims to identify the most active and productive authors and their collaborators, visualize collaboration intensity, and detect influential research teams and potential collaborators, thereby facilitating the development of stronger collaborative networks (Xia et al., 2022). Based on the VOSviewer-generated co-authorship network (threshold ≥5 publications), the author network comprises two primary collaborative clusters alongside several smaller, isolated sub-networks (components). The primary clusters are led by highly productive authors including Nahrendorf, Matthias, Egashira, Kensuke, and Matoba, Tetsuya (14 publications each). This overall fragmented topology indicates that research in this field is predominantly organized around independent research groups with localized collaboration. Overall, although China ranks first globally in terms of total publication output in this field, the most productive and highly co-cited authors are predominantly from the United States and Japan. This pattern may be attributed to the substantial foundational research conducted by scholars in these countries, particularly during the early stages of the field, which laid a strong academic groundwork for subsequent developments.

### 3.6 Analysis of references

The top ten most cited references are listed in Table 4. The most cited paper is the review article published by Zhao T. et al. (2022) in the journal *Bioactive Materials* (Zhao T. et al., 2022). This study systematically elaborated on the pathological mechanism of myocardial ischemia-reperfusion injury (IRI), and focused on the core role of excessive reactive oxygen species (ROS) generation in triggering myocardial cell apoptosis and exacerbating inflammatory responses. The authors particularly pointed out the limitations of traditional antioxidant therapies, such as short half-life, low bioavailability, and insufficient targeting.

**TABLE 4:** The top 10 co-cited references of nanomaterials and CHD research.

Rank	Title	Journal	Author	Citations	Centrality
1	Reactive oxygen species-based nanomaterials for the treatment of myocardial ischemia reperfusion injuries	*Bioactive Materials*	Tianjiao Zhao	18	0.01
2	Minimal information for studies of extracellular vesicles 2018 (MISEV 2018): a position statement of the International Society for Extracellular Vesicles and update of the MISEV2014 guidelines	*Journal of Extracellular Vesicles*	Clotilde Théry	17	0.11
3	Drug-loaded multifunctional nanoparticles targeted to the Endocardial Layer of the Injured heart modulate Hypertrophic Signaling	*Small*	Mónica P A Ferreira	16	0.18
4	Heart disease and Stroke Statistics-2017 update: A Report from the American heart Association	*Circulation*	Emelia J Benjamin	16	0.01
5	Nanoparticle-mediated delivery of pitavastatin inhibits atherosclerotic plaque destabilization/rupture in mice by regulating the recruitment of inflammatory monocytes	*Circulation*	Shunsuke Katsuki	15	0.14
6	Engineering precision nanoparticles for drug delivery	*Nature Reviews Drug Discovery*	Michael J Mitchell	15	0.11
7	Myocardial ischaemia-reperfusion injury and cardioprotection in perspective	*Nature Reviews Cardiology*	Gerd Heusch	15	0.01
8	Signaling pathways and targeted therapy for myocardial infarction	*Signal Transduction And Targeted Therapy*	Qing Zhang	15	0.01
9	Leukocyte behavior in atherosclerosis, myocardial infarction, and heart failure	*Science*	Filip K Swirski	14	0.13
10	VEGF nanoparticles repair the heart after myocardial infarction	*American Journal of Physiology-heart And Circulatory Physiology*	Yasin Oduk	14	0.07

The review also discussed emerging ROS-responsive nanomedicine strategies, including the use of nanoenzymes and drug-loaded nanoparticles to reshape the oxidative-reductive microenvironment of the myocardium, regulate mitochondrial function, and intervene in macrophage polarization, providing an important theoretical basis for nanomaterial-based antioxidant therapies for the intervention of antioxidant and anti-inflammatory treatment of CVDs with nanomaterials. Currently, the nanotherapeutic strategies for CVDs have extended from theoretical mechanisms to highly specific targeted delivery system design. In terms of stabilizing atherosclerotic plaques, Katsuki et al. (2014) successfully constructed poly(lactic-co-glycolic acid) nanoparticles loaded with pitavastatin. Experiments showed that this system could be efficiently taken up by circulating inflammatory monocytes (Ly-6G^−^CD11b^+^) through intravenous injection. In the mouse model, this nanosystem significantly reduced the infiltration of monocytes and the activity of gelatinase (MMP-9) in the plaque, effectively inhibiting plaque instability and rupture, verifying the delivery potential targeting inflammatory cells.

In the aspects of local remodeling and targeted therapy after myocardial ischemia, Ferreira et al. (2017) developed multifunctional nanocarriers made of porous silicon modified with atrial natriuretic peptide. This study utilized the characteristic of the damaged myocardial endocardium layer expressing high levels of natriuretic peptide receptors, achieving efficient “homing” of nanomedicines to the left ventricular endocardium. The heart-protective drug (C1) carried by this system successfully reduced the myocardial hypertrophy signal driven by ERK1/2 *in vivo*, demonstrating excellent performance in precisely targeting vulnerable myocardial regions. While exploring synthetic nanocarriers, natural biomimetic nanodelivery systems represented by EVs have also increasingly gained attention in myocardial repair. To standardize the quality control in this cutting-edge field, Théry et al. (2018) represented the International Society for Extracellular Vesicles and released a landmark guideline. This guideline systematically defines the nomenclature of EVs from both theoretical and technical perspectives, and establishes rigorous experimental standards for isolation, quantification, protein/lipid characterization, and functional validation. This consensus provides an crucial regulatory foundation for clarifying the biological functions of various subgroups of vesicles and ensuring the data reproducibility and clinical translation of natural biomimetic nanocarriers in cardiovascular disease models. Using the Citespace software to draw a reference literature emergent graph, one can identify the literature that relevant researchers have extensively focused on within a certain period (Wu et al., 2022).

Figure 6A presents the top 50 references with the highest citation frequency. Among them, the reference with the highest citation frequency is the paper “Therapeutic siRNA silencing in murine inflammatory monocytes” published by Florian Leuschner et al. in the journal *Nature Biotechnology* (2013–2016, with a citation intensity of 7.27) (Leuschner et al., 2011). Additionally, the articles by Libby et al. (2019), Tan et al. (2021) are recent highly-cited references with citation emergence phenomena(Figure 7A).

**FIGURE 6:**
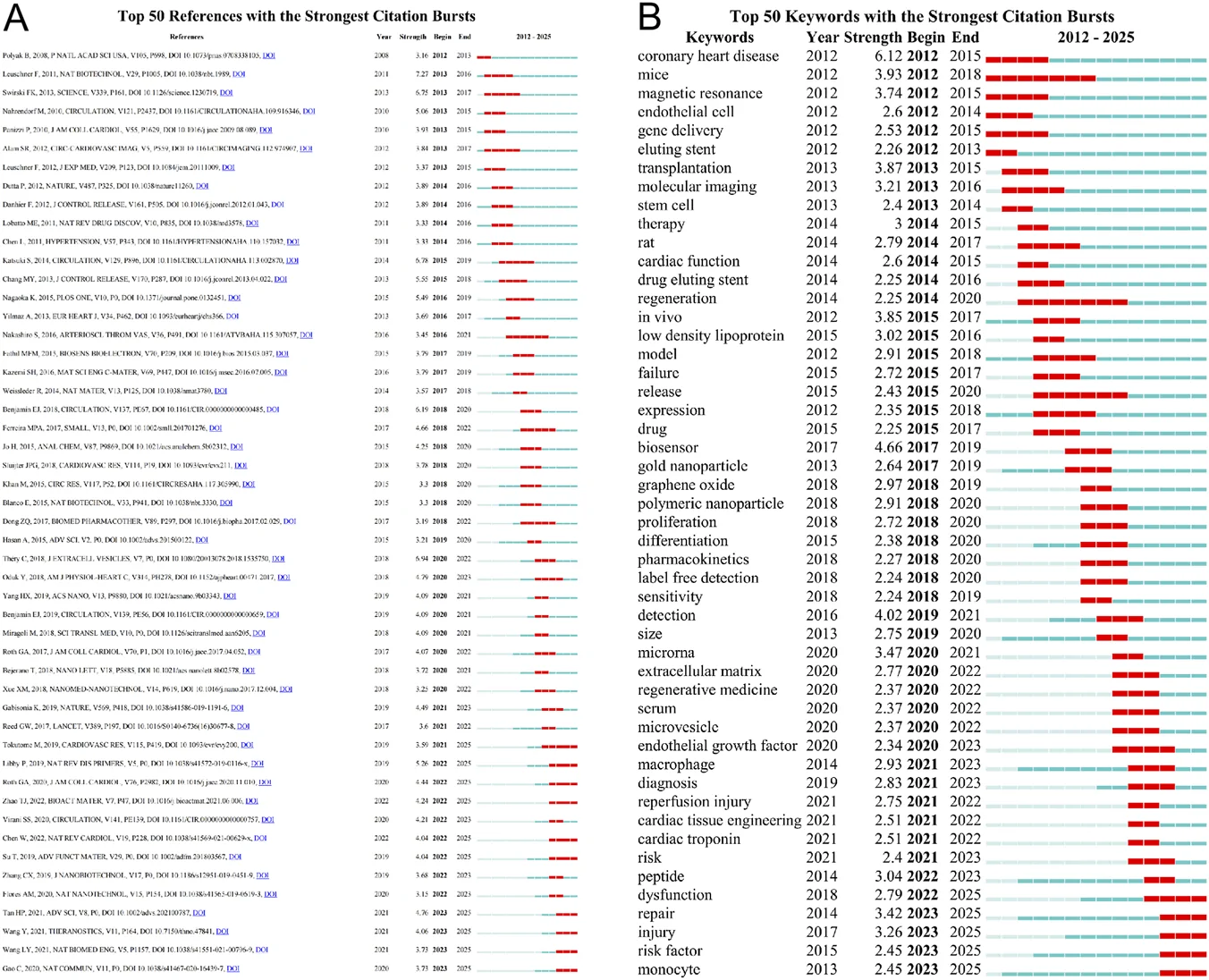
Citation burst and keyword burst analysis **(A)** Bursting map of cited literatures. **(B)** Bursting map of keywords.

**FIGURE 7:**
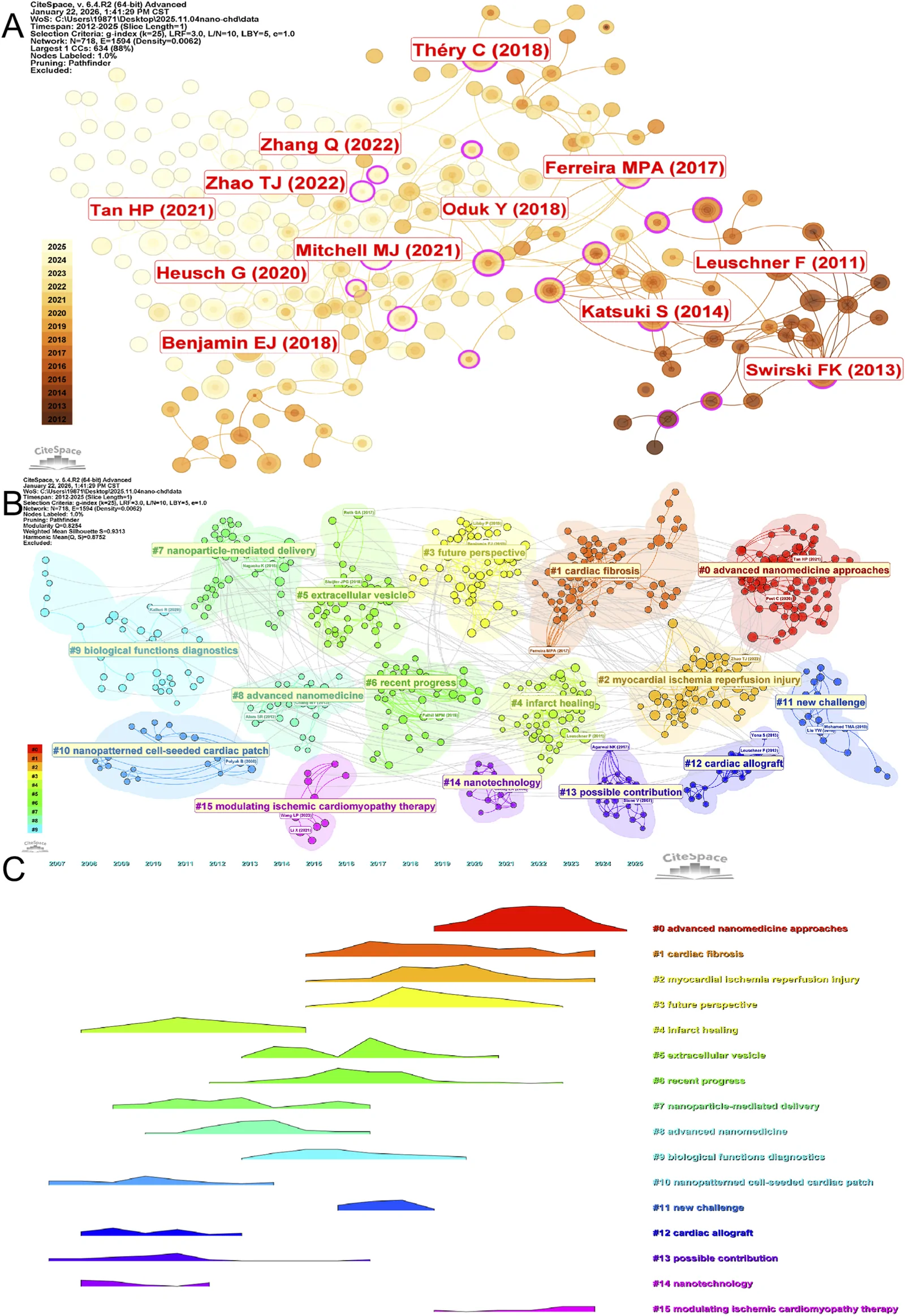
Co-citation analysis of references. **(A)** Co-citation network of references. **(B)** Clustered co-citation network highlighting major thematic groups. **(C)** Temporal evolution of co-citation clusters.

To further clarify the knowledge structure and evolution trajectory of the field related to CHD and nanomaterials, a cluster analysis and landscape view (Figures 7B,C) were conducted on the co-cited references. The results clearly present the temporal evolution of research focus and the development stages of themes: In the early period (2007–2015), the research mainly focused on basic exploration and macro-scale tissue engineering intervention, focusing on #10 nano-patterned cell inoculation of heart patches, #4 infarction healing, #12 heart allograft transplantation, and #14 nano-technology basic applications, laying the foundation for the transformation of nanoscience to ischemic heart disease intervention; in the mid-period (2010–2017), the research direction shifted to microscopic functional delivery systems, mainly focusing on #7 nanoparticle-mediated delivery, #5 extracellular vesicles, and #9 biological functions and diagnostics, emphasizing the design of targeted carriers and the analysis of mechanism of action; in the recent period (2018 to present), it has moved towards precise nanomedicine targeting specific pathological mechanisms, with #0 advanced nanomedicine methods becoming the core theme, and #2 myocardial ischemia reperfusion injury, #1 myocardial fibrosis, and #15 ischemic cardiomyopathy treatment regulation, etc., directions continuously deepening, representing the frontier and future trends of the field. Overall, this field presents a clear technological evolution path from macro-scale tissue engineering and basic nanotechnology to multi-functional nanodelivery systems, and then to precise regulation of pathological microenvironments, achieving a transformation from material-centered to pathology-driven precise intervention, thereby strengthening the reliability of the literature metrics findings of this study.

### 3.7 Analysis of journals

A total of 2,709 publications in this field were distributed across 726 journals, among which 116 journals published five or more articles. As shown in Table 5, *Biomaterials* ranks first in terms of publication output (n = 88), followed by *Biosensors and Bioelectronics* (n = 63) and *Journal of Controlled Release* (n = 57). Figures 8A,B, together with Table 5, present the top 10 journals ranked by publication output and co-citation frequency. Among the 6,242 co-cited journals, 62 journals received more than 200 citations. *Circulation* (Impact Factor: 38.73, Q1) ranks first in co-citation frequency, followed by *Circulation Research* (IF: 16.2, Q1), *Journal of the American College of Cardiology* (IF: 22.3, Q1), and *Biomaterials* (IF: 12.9, Q1). Among the top 10 co-cited journals, nine belong to the Q1 category of the JCR, and eight have an impact factor greater than 5. While journal-level metrics (e.g., IF and quartile rank) do not directly establish the intrinsic scientific quality of individual co-cited articles, this distribution reflects the high academic visibility, rigorous peer review, and broad dissemination reach of core cardiovascular nanomedicine research within authoritative venues.

**TABLE 5:** The top 10 most productive journals and co-cited journals.

Rank	Journals	Counts	TLS	If and JCR division (2025)	Co-cited journals	Total number of co-citations	If and JCR division (2025)
1	*Biomaterials*	88	170	12.9, Q1	*Circulation*	570	38.73, Q1
2	*Biosensors and bioelectronics*	63	85	16.1, Q1	*Circulation Research*	434	16.2, Q1
3	*Journal of controlled release*	57	103	13.3, Q1	*Journal of the American College of Cardiology*	421	22.3, Q1
4	*Sensors and actuators b: chemical*	55	49	6.5, Q1	*Biomaterials*	416	12.9, Q1
5	*Chemical engineering journal*	55	39	13.3, Q1	*Acs Nano*	383	16.1, Q1
6	*Dental materials*	48	4	8.2, Q1	*Proceedings of the National Academy of Sciences of the United States of America*	371	9.1, Q1
7	*Acta biomaterialia*	46	52	11.5, Q1	*Journal of Controlled Release*	352	11.5, Q1
8	*International journal of biological macromolecules*	40	16	12.1, Q1	*Nature*	343	48.5, Q1
9	*the journal of prosthetic dentistry*	40	6	19, Q1	*Scientific Reports*	323	3.9, Q1
10	*Bioactive materials*	37	70	9.6, Q1	*PloS One*	313	2.6, Q2

**FIGURE 8:**
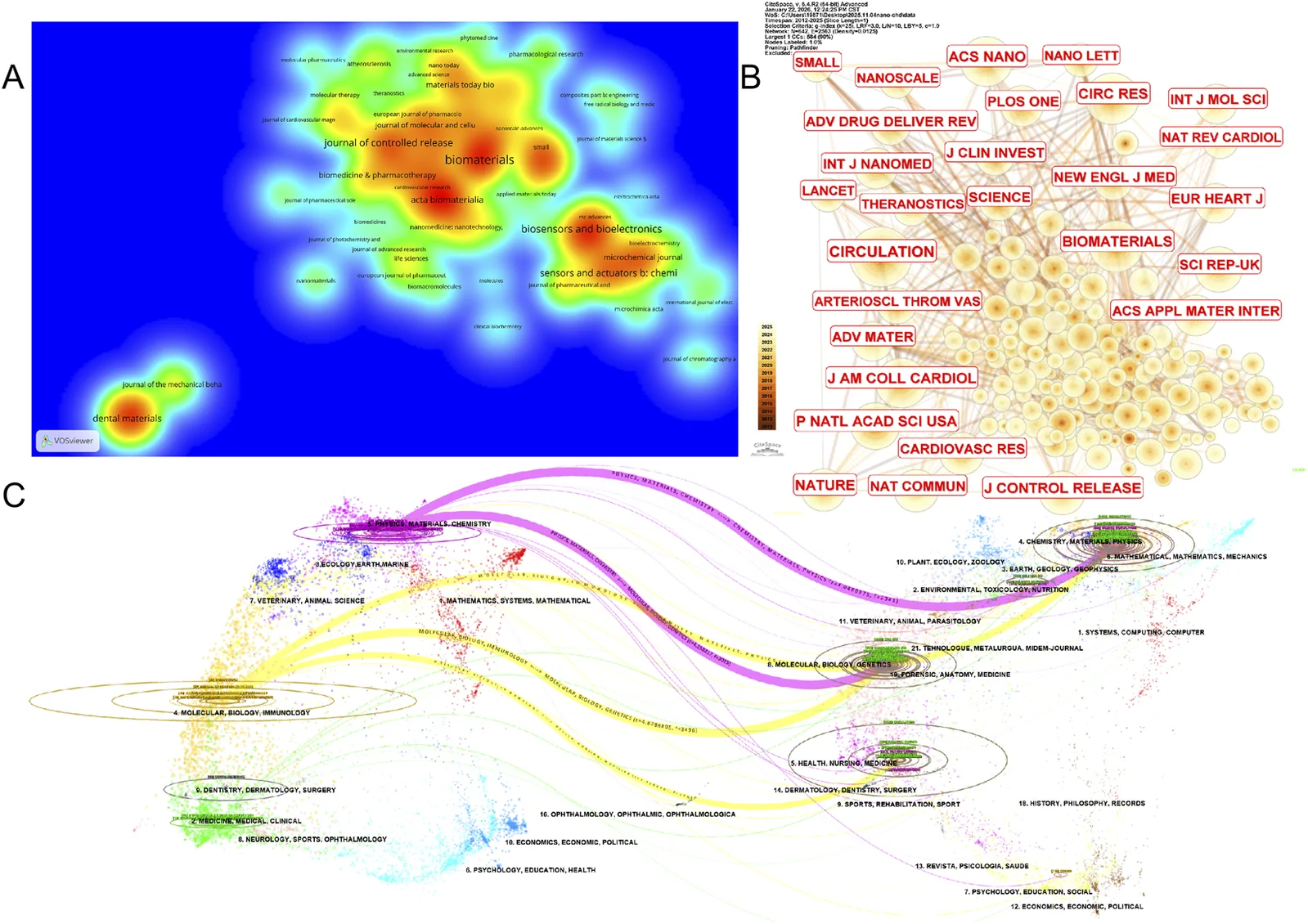
Analysis of journals. **(A)** Density map of journal publications; **(B)** Co-citation network map of journals; **(C)** dual map of journals: The ellipse represents the number of publications associated with each journal. The length of the horizontal axis reflects the number of contributing authors; The length of the vertical axis represents the number of publications.

The dual-map overlay of journals (Figure 8C) illustrates the flow of knowledge within this research domain. In this visualization, citing journals are presented on the left, while cited journals appear on the right, and the colored citation paths represent the directional flow of knowledge (Ma et al., 2021; Jiang et al., 2025). Five primary citation pathways are identified, indicating that studies published in journals related to Molecular/Biological/Immunological and Physics/Materials/Chemistry fields predominantly cite journals in the domains of Molecular/Biology/Genetics, Chemistry/Materials/Physics, and Health/Nursing/Medicine. This complex citation pattern highlights the pronounced interdisciplinary nature of the field, reflecting extensive integration and knowledge exchange among fundamental life sciences, materials science, and clinical medicine.

### 3.8 Analysis of keywords and research hotspots in CHD nanomedicine

Keywords effectively summarize the core content of publications, and keyword co-occurrence analysis serves as a valuable approach for identifying research hotspots in the field of CHD-related nanomaterials. The most frequently occurring keyword was “myocardial infarction”, while “coronary heart disease” exhibited the highest betweenness centrality. Other keywords with high centrality included “*in vitro*”, “heart failure”, and “magnetic resonance” (Figure 9A; Table 6). After removing irrelevant terms, VOSviewer was employed to construct a keyword co-occurrence network comprising 104 keywords with a minimum occurrence threshold of 20. This analysis resulted in six distinct clusters (Figure 9B; Supplementary Figure S1). Cluster 1 (red) included 26 keywords and primarily focused on myocardial repair and regenerative medicine. Representative terms included MI, cardiac repair, cardiac regeneration, myocardial cells, mesenchymal stem cells, stem cells, exosomes, extracellular vesicles, hydrogels, injectable hydrogels, lipid nanoparticles, gene therapy, and microRNA. Cluster 2 (green) comprised 19 keywords and centered on nanomedicine-based therapeutic strategies and drug delivery systems for CVDs, including CVDs, coronary artery disease, drug delivery, targeted delivery, nanomedicine, nanocarriers, liposomes, magnetic nanoparticles, mesoporous silica nanoparticles, pharmacokinetics, and *in vitro* studies. Cluster 3 (blue) contained 18 keywords and focused on early diagnosis and biosensing technologies for myocardial injury, including acute myocardial infarction (AMI), biomarkers, cardiac troponin, myoglobin, C-reactive protein, biosensors, aptamer-based sensors, immunoassays, gold nanoparticles, and graphene oxide. Cluster 4 (yellow) also included 18 keywords and primarily addressed the pathophysiological mechanisms of myocardial ischemia–reperfusion injury, such as ischemia–reperfusion injury, ROS, OS, apoptosis, autophagy, nitric oxide(NO), release, activation, inhibition, and cardioprotection. Cluster 5 (purple) consisted of 17 keywords and emphasized *in vivo* imaging and the regulation of AS and inflammatory microenvironments, including AS, thrombosis formation, macrophages, monocytes, inflammation, fibrosis, molecular imaging, MRI, contrast agents, and *in vivo* studies. Cluster 6 (light blue) included 8 keywords and focused on advanced biomaterials and 3D printing technologies in tissue engineering, such as tissue engineering, regenerative medicine, biomaterials, scaffolds, 3D printing, additive manufacturing, and CAD/CAM. A cluster timeline visualization generated using CiteSpace further illustrated the temporal evolution of research hotspots (Figure 9C; Table 7). The keyword clustering network yielded a modularity Q value of 0.4436, exceeding the recommended threshold of 0.3, which indicates a well-defined clustering structure. The mean silhouette score was 0.7639, suggesting high internal consistency and clear separation among clusters. Furthermore, representative keywords within each cluster were manually reviewed to confirm their biological and epidemiological relevance to the field, thereby enhancing the interpretability and robustness of the clustering results.

**FIGURE 9:**
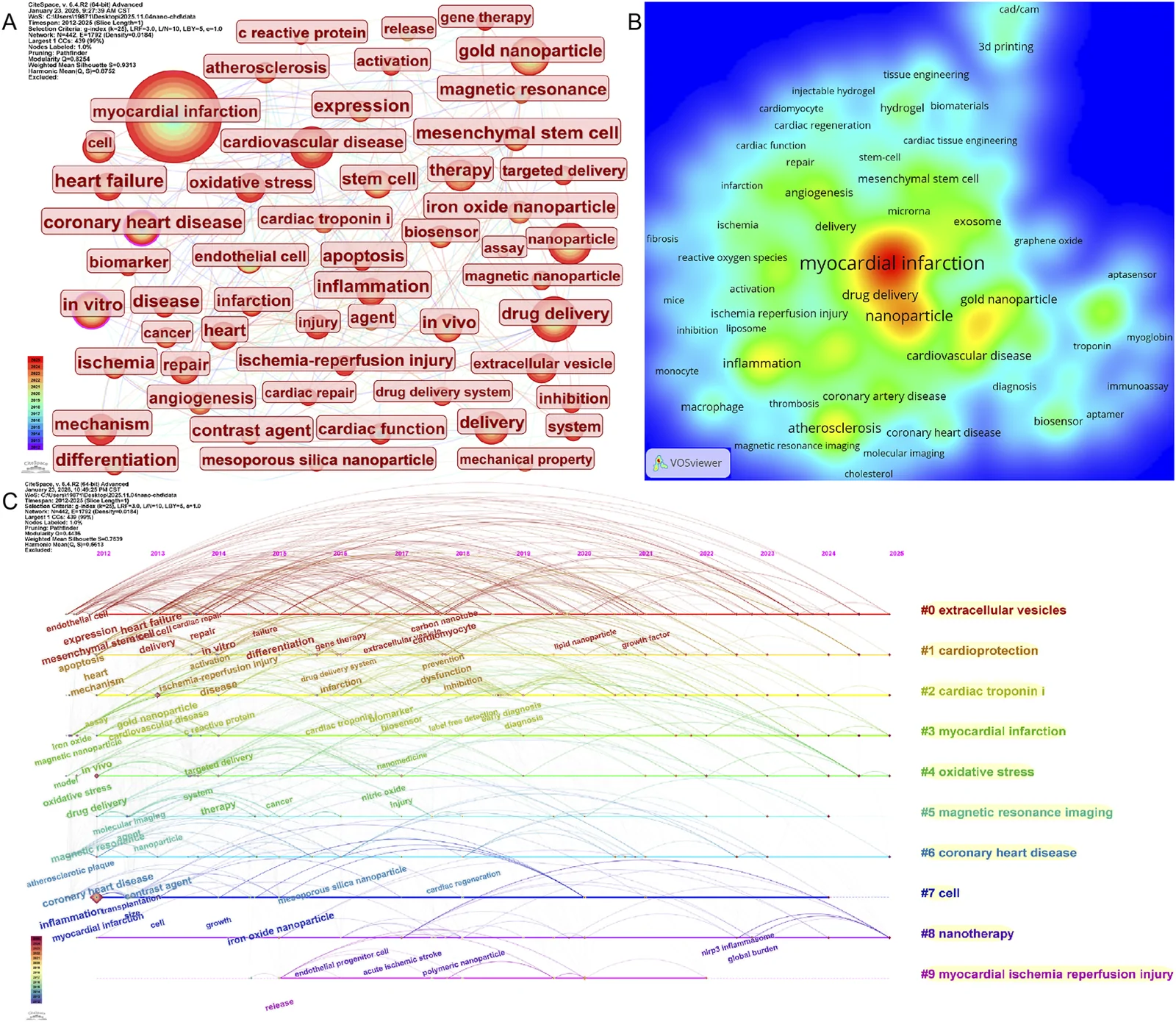
Keyword analysis. **(A)** Co-occurrence of keywords. **(B)** Keyword density map. **(C)** Timeline view of keywords cluster.

**TABLE 6:** Top 10 keywords by frequency and centrality in the field of nanomaterials and CHD.

Rank	Keywords	Frequency	Keywords	Centrality
1	Myocardial infarction	429	Coronary heart disease	0.12
2	Drug delivery	121	*In vitro*	0.1
3	Nanoparticle	108	Heart failure	0.1
4	Cardiovascular disease	91	Magnetic resonance	0.1
5	Gold nanoparticle	88	Inflammation	0.09
6	*In vitro*	72	Drug delivery	0.08
7	Delivery	71	Therapy	0.08
8	Cell	70	*In vivo*	0.08
9	Coronary heart disease	69	Mesenchymal stem cell	0.08
10	Inflammation	66	Disease	0.08

**TABLE 7:** Main research directions and representative keywords in the field of nanomaterials and CHD.

No.	Research direction	Cluster ID	Representative keywords(LLR)
1	Nano-biosensing and molecular Imaging	#2, #5	Gold nanoparticle, biosensor, Immunoassay, cardiac troponin I, cardiac biomarkers, magnetic resonance Imaging, molecular Imaging, collagen, Photocatalysis, myocardial infarction
2	Cardioprotection and microenvironment modulation	#1, #4, #9	Myocardial ischemia reperfusion injury, nanozymes, nanoformulation, ROS, OS, NO, bioelectronics, pharmacokinetics, cardioprotection, therapeutic neovascularization
3	Targeted drug delivery and precision Nanotherapy	#3, #6, #8	High density lipoprotein, Chitosan nanoparticles, Heparin, RNAi, thrombolysis, Stroke, photodynamic therapy, precision medicine, pathway, post-infarction ventricular remodelling
4	Regenerative medicine and Cellular Engineering	#0, #7	Heart failure, Stem cells, Extracellular vesicles, tissue engineering, angiogenesis, cell, Endocytosis

Keyword bursts refer to terms that exhibit a sudden increase in frequency over a specific period, thereby facilitating the identification of emerging research frontiers and reflecting evolving trends within a field. Figure 6B presents the top 50 keywords with the strongest burst intensities. Among these, “coronary heart disease” demonstrates the highest burst strength (intensity = 6.12, 2012–2015), highlighting the initially well-defined core disease focus of this research domain. The developmental trajectory of this field can be broadly divided into two stages. The first stage (2012–2017) is characterized by macroscopic and structure-oriented research, with keywords primarily related to fundamental diagnosis and interventional treatment, including magnetic resonance, drug-eluting stents, and gene delivery. The sustained burst of animal model–related keywords, such as “mice” and “rat”, indicates active advancement in preclinical studies during this period. The second stage (2018–2025) reflects a shift toward micro-level exploration, functionalization, and diversification. In the context of early diagnosis, “biosensor” and “cardiac troponin” emerged as major research hotspots. In the area of targeted delivery, gold nanoparticles, polymeric nanoparticles, and pharmacokinetics have continued to receive sustained attention. Meanwhile, in terms of pathological mechanisms and repair strategies, keywords such as dysfunction, injury, repair, macrophage, and monocyte have shown persistent burst signals since 2020 and remain prominent research directions. In addition, frontier topics such as regenerative medicine and cardiac tissue engineering have exhibited strong burst intensities since 2020. Together with broader concepts such as risk factors, these findings suggest that research in this field is gradually expanding toward a more integrated landscape, incorporating risk assessment, molecular regulatory mechanisms, and tissue regeneration strategies.

## 4 Discussion

### 4.1 Overall distribution

CHD has emerged as an increasingly severe global public health crisis, driven by population aging and the widespread adoption of unhealthy lifestyles, and it remains the leading cause of mortality worldwide (GBD 2019 Diseases and Injuries Collaborators, 2020). Myocardial ischemia and its associated secondary injuries exert a profound impact on patient prognosis, imposing a substantial socioeconomic burden on a global scale. Despite continuous advances in cardiovascular management, conventional therapeutic strategies—including standard pharmacotherapy and invasive revascularization—primarily focus on acute reperfusion and secondary event reduction. However, their capacity to directly attenuate adverse post-infarction remodeling or stimulate myocardial regeneration remains constrained by systemic off-target effects and limited target-site bioavailability. These approaches not only fail to effectively repair damaged cardiomyocytes but also lack disease-modifying strategies capable of fundamentally reversing the local inflammatory and oxidative stress(OS) microenvironment (Deedwania and Carbajal, 2011). To overcome this clinical bottleneck, nanomaterials—owing to their superior targeting capabilities toward pathological microenvironments, stimuli-responsive drug delivery properties, and enhanced bioavailability—have emerged as a promising frontier in CHD-targeted therapy. In this study, a bibliometric analysis was conducted to systematically evaluate the global research landscape of nanomaterials in CHD treatment from 2012 to 2025, with particular emphasis on publication trends, collaboration networks, research hotspots, and translational potential. These findings provide critical insights into the evolutionary trajectory of nanotechnology in CHD diagnosis and therapy and offer valuable guidance for future interdisciplinary research. Over the past decade, the exponential growth in related scholarly output highlights the rapidly increasing interest in nanomaterials within the cardiovascular field. This growing focus on nanotechnology-driven solutions aims to address key limitations of conventional CHD therapies, including low drug delivery efficiency, significant systemic side effects, and the lack of effective strategies to reverse disease progression—such as promoting atherosclerotic plaque regression or repairing damaged myocardium. The surge in academic output can largely be attributed to advances in materials science, which have enabled the development of intelligent, stimuli-responsive nanocarriers capable of precise delivery within diverse cardiovascular pathological microenvironments, including those characterized by elevated ROS, inflammation, and mild acidity. These innovations have facilitated the emergence of novel therapeutic strategies with prolonged target-site retention and enhanced specificity. Simultaneously, the growing clinical demand for improved CHD management—particularly for precision therapies with reduced adverse effects and sustained efficacy—is being progressively addressed through innovative applications of nanomaterials in controlled drug release, gene therapy, and vascular endothelial and myocardial tissue repair.

From a geographical perspective, research on nanomedicine for CHD is highly concentrated in a limited number of technologically advanced countries. China ranks first in publication volume, with 402 articles (accounting for 14.84%), supported by strong governmental policies and funding, and hosts seven of the world’s top ten most productive institutions, reflecting its substantial research capacity. In contrast, although the United States ranks second in publication output (210 articles), it maintains a leading position in terms of total citations (11,529), average citations per paper (54.90), and network centrality (0.58). Key institutions in this field include Harvard University, the Chinese Academy of Sciences, and Massachusetts General Hospital. Although China–US collaboration is relatively active, international cooperation remains concentrated among major research-producing countries, indicating the need for broader cross-regional collaboration to facilitate knowledge exchange and clinical translation. Regarding influential scholars, Nahrendorf, M. leads in productivity with 14 publications and a high total citation count, while Libby, P. ranks first in the co-citation network with 125 citations. Together, they constitute a central driving force in advancing knowledge development in this field.

The dual-map overlay of journals, along with high-output journals such as *Biomaterials* and highly co-cited journals such as *Circulation*, underscores the inherently interdisciplinary nature of this research domain. Its knowledge base integrates theories from molecular biology, genetics, chemistry, materials science, physics, and clinical medicine. Meanwhile, frontier research is primarily driven by journals in molecular biology, immunology, and materials science, clearly delineating the translational pathway from fundamental materials science to cardiovascular clinical applications. The following section will briefly outline key studies and future research directions in this field.

### 4.2 Research hotspots

#### 4.2.1 Nano-biosensing and molecular imaging

Accurate and rapid early diagnosis is a prerequisite for preventing fatal complications of MI (Xie et al., 2025). However, conventional clinical detection methods face dual limitations in sensitivity and timeliness, particularly in the identification of low-abundance biomarkers (Zhang et al., 2020). In recent years, biosensors and immunoassays based on advanced nanomaterials, such as gold nanoparticles (AuNPs), have markedly improved *in vitro* screening sensitivity. Early detection of high-sensitivity cardiac troponin (cTn) remains central to MI diagnosis, and studies have demonstrated its high diagnostic accuracy even in elderly populations (Reiter et al., 2011). When combined with additional biomarkers, such as copeptin, the early diagnostic rate within 3 h of chest pain onset can be significantly enhanced (Keller et al., 2010). To further advance diagnostic performance, a variety of innovative nanoplatforms have been developed. For instance, In_2_O_3_ nanobelt-based biosensors enable rapid detection of biomarkers with ultrahigh sensitivity, achieving limits well below clinical cutoff concentrations (Liu et al., 2016). Similarly, plasmonic chips based on gold nanoislands exhibit superior sensitivity and specificity compared with conventional chemiluminescence immunoassays (Xu et al., 2020). In addition, electrochemical dual-aptamer sensors (Sun et al., 2019a; Sun et al., 2019b), multifunctional gold nanoparticles (Li et al., 2020), microfluidic paper-based analytical devices (Lim et al., 2019), and dynamic enzyme-linked immunosorbent assays (Li D. et al., 2021) have enabled the simultaneous, highly sensitive detection of multiple biomarkers within a very short time frame, thereby facilitating early and rapid diagnosis of AMI.

Breakthroughs in ultrasensitive *in vitro* diagnosis of AMI are fundamentally driven by the incorporation of nanomaterial-enabled physicochemical properties, including surface plasmon resonance (SPR), localized surface plasmon resonance (LSPR), and photocatalytic effects. These mechanisms markedly enhance signal amplification at the sensing interface, thereby enabling highly sensitive quantification of trace myocardial biomarkers, such as cardiac troponin I (cTnI) (Zeng et al., 2014). For instance, SPR biosensors amplified by gold nanoparticles (AuNPs) have achieved limits of detection as low as 10 pM for cTnI, allowing not only precise identification of pathological abnormalities but also dynamic monitoring of subtle physiological fluctuations (Gnedenko et al., 2013; Ranjbari et al., 2024). Moreover, functionalization of AuNP surfaces with specific recognition probes enables target binding to directly modulate the local refractive index, resulting in a redshift of the LSPR spectrum. This label-free detection strategy eliminates complex labeling procedures, reduces operational costs, and demonstrates high diagnostic specificity in complex biological matrices (Libby, 2021). The convergence of these nanotechnologies provides robust theoretical and technical support for early intervention in MI (Khan et al., 2020; Fan P. et al., 2025; Ge et al., 2025). Concurrently with advances in ultrasensitive *in vitro* screening, progress in in vivo visualization—particularly in molecular imaging and MRI—has enabled precise localization of pathological lesions. Emerging nano-contrast agents can selectively bind to collagen networks or inflammatory macrophages within atherosclerotic plaques, facilitating real-time *in situ* imaging. For example, sulfated dextran-coated iron oxide nanoparticles specifically target macrophage scavenger receptor class A, significantly enhancing preferential uptake and sensitivity in plaque MRI (Tu et al., 2011). In addition, AuNP-based Single-Photon Emission Computed Tomography/Computed Tomography imaging probes enable accurate assessment of apoptotic macrophages within lesions (Li et al., 2016). To address other complex pathological targets, such as collagen-rich matrices, dual-modal Fe_3_O_4_ nanoprobes targeting osteopontin -overexpressing foam macrophages (Qiao et al., 2017), as well as aggregation-induced emission nanoprobes functionalized with anti-CD47 antibodies via modulation of rhodanine substituents (Wang et al., 2022), have demonstrated outstanding precision in the noninvasive detection of vulnerable plaques. This seamless integration of ultrasensitive *in vitro* biosensing with *in vivo* pathological imaging is transforming CHD management from macroscopic symptom-based intervention toward precise early warning at the subclinical stage (Herkert and Garcia-Parajo, 2025).

#### 4.2.2 Cardioprotection and microenvironment modulation

During revascularization in CHD-associated AMI, the restoration of blood perfusion paradoxically triggers myocardial ischemia–reperfusion injury (IRI), which substantially limits cardiac functional recovery in critically ill patients (Wang et al., 2020). Within this pathological cascade, the excessive burst of ROS and sustained OS in the damaged myocardium constitute key drivers of secondary cardiomyocyte apoptosis and coronary microvascular dysfunction (Yang et al., 2022; Xiang et al., 2023). To intercept this biochemical damage at its source, nanozymes with intrinsic catalytic activity and intelligent nanoformulations have demonstrated considerable therapeutic potential. These emerging nanomaterials can mimic endogenous antioxidant enzyme systems to selectively scavenge excessive free radicals and restore NO metabolic homeostasis. For example, in response to the acidic microenvironment of ischemic tissue, an acid-triggered cascade-responsive supramolecular peptide has been developed, which effectively attenuates ROS bursts and suppresses apoptosis by re-establishing redox balance (Liao et al., 2025). Further advances include mitochondria-targeted nanozymes engineered via genetic approaches, which not only catalyze the conversion of L-arginine to NO to ameliorate endothelial dysfunction but also reduce mitochondrial ROS levels and restore mitochondrial membrane potential (Zhang X. et al., 2025). Such organelle-targeted antioxidant and endothelial-protective strategies provide a robust foundation for remodeling the biochemical microenvironment of ischemic myocardium. Beyond mitigating endothelial injury and redox imbalance, reconstruction of the disrupted physical and electrophysiological conduction network within infarcted regions represents another critical avenue for achieving substantive cardioprotection. In recent years, conductive nanomaterials integrated with bioelectronic design principles have gained increasing attention in cardiac tissue engineering. These materials can provide electrical conductivity comparable to native myocardium and, through their unique electrical, mechanical, and topological properties, not only precisely regulate cardiomyocyte phenotype, growth, and differentiation *in vitro* (Ryan et al., 2018; Ashtari et al., 2019), but also promote the restoration of electromechanical function *in vivo* (Morsink et al., 2022). For instance, conductive nanocomposite hydrogels, owing to their excellent electrical conductivity and biomimetic matrix characteristics, can effectively bridge disrupted myocardial electrical conduction pathways, significantly reducing cardiomyocyte death and pathological fibrosis (Lee et al., 2022; Zhao G. et al., 2022; Liu et al., 2025). In addition, the integration of nanomaterials with biomacromolecules such as collagen has enabled the development of ionically conductive cardiac patches with electrophysiological responsiveness. These platforms not only markedly improve cardiac systolic and diastolic function *in vivo* (Hosoyama et al., 2018), but also establish large-area, stable, and flexible bioelectronic interfaces for high-resolution electrophysiological mapping, real-time monitoring, and targeted feedback stimulation during MI progression (Shen et al., 2023; Han et al., 2024). The convergence of microenvironment modulation and electrophysiological repair, when combined with advanced drug delivery systems, ultimately forms a comprehensive cardioprotective network. In the context of the complex infarct microenvironment, nanoparticle-based systems—with their precise spatiotemporal control over drug release—can significantly improve the pharmacokinetics of therapeutic agents, protecting them from enzymatic degradation while prolonging retention within the injured myocardial target site (Fan et al., 2020). Notably, the integration of intelligent drug delivery strategies with the matrix advantages of conductive nanomaterials not only substantially improves cardioprotective efficacy but also provides critical mechanical support to damaged tissues (Kai et al., 2013; Wang et al., 2017; Morsink et al., 2022). From eliminating ROS-driven cascade damage at its origin to correcting local endothelial and electrophysiological dysfunction, nanomedicine achieves multi-level remodeling of the pathological microenvironment, thereby overcoming the limitations of conventional single-target therapies and establishing an optimal tissue matrix for subsequent therapeutic angiogenesis (Steinhoff et al., 2017).

#### 4.2.3 Targeted drug delivery and precision nanotherapy

In the clinical management of CHD, conventional systemic drug administration is often constrained by low bioavailability, increased bleeding risk, and significant off-target effects. To overcome these intrinsic physiological barriers, therapeutic strategies are rapidly evolving toward precision nanomedicine. The core concept lies in the use of highly engineered biomimetic nanocarriers—such as high-density lipoprotein (HDL)-mimetic nanoparticles, platelet membrane-coated nanocarriers, and microenvironment-responsive platforms—to achieve targeted accumulation and lesion-specific homing of therapeutic agents within atherosclerotic plaques or ischemic myocardial regions. Specifically, HDL-mimetic nanoplatforms exhibit high affinity for macrophages within plaques and can effectively modulate their polarization states, thereby regulating inflammatory responses and lipid metabolic dysfunction (Zhen et al., 2024; He et al., 2025). Platelet membrane-based biomimetic carriers demonstrate unique advantages in addressing complex vascular pathologies. For example, a deformable platelet-mimicking delivery system can physically adapt to the heterogeneous porous architecture of thrombi, enabling the co-delivery of urokinase and atorvastatin to achieve targeted thrombolysis and sustained endothelial repair (Zhang J. et al., 2025). Furthermore, leveraging the intrinsic multivalent affinity of platelet membranes, these biomimetic systems can precisely anchor to activated endothelial cells and foam cells, facilitating efficient localization and early intervention of vulnerable plaques at the subclinical stage (Wei et al., 2018). To further overcome barriers related to deep penetration and retention within the complex plaque microenvironment, advanced strategies incorporating stimuli-responsive mechanisms and receptor-specific modifications have been developed to enable synergistic theranostic interventions. For instance, targeting the acidic and ROS-enriched microenvironment of plaques, an intelligent responsive nanoplatform (PA/ASePSD nanoparticles), integrating π-conjugated polymers with astaxanthin, not only actively targets damaged endothelium via a dextran shell but also undergoes cascade disassembly upon microenvironmental stimulation. This system enables high-contrast, noninvasive photoacoustic imaging while simultaneously promoting anti-inflammatory effects and lipid metabolism regulation through upregulation of cholesterol efflux receptors (Xu et al., 2023).

Similarly, the LAID dual-modal imaging platform undergoes disassembly under oxidative and acidic conditions, enabling precise targeting of lipid cores and suppression of plaque progression via inhibition of the NF-κB signaling pathway (Wang et al., 2024). In terms of deep tissue targeting, transferrin (Tf)-modified nanocapsules facilitate delivery of therapeutic agents up to 70 μm into three-dimensional tissue structures via enhanced transcytosis (Kohata et al., 2019). Meanwhile, melanin-based theranostic probes coated with inflammation-homing neutrophils not only aggregate in response to matrix metalloproteinase (MMP) activation within plaques—prolonging retention time—but also reduce macrophage burden and lipid accumulation through intrinsic ROS scavenging properties (Fan B. et al., 2025). Such microenvironment-regulating strategies based on precision nanodelivery provide essential support for the safe translation of labile therapeutic agents—including NO, RNA interference molecules, and photosensitizers—and enable multimodal combination therapies, ultimately achieving comprehensive “disease modification” in CHD. For example, S-nitrosylated phospholipid–ApoA-I biomimetic nanoparticles effectively overcome the ultrashort half-life of NO, significantly alleviating ischemia–reperfusion injury and reducing plaque burden (Rink et al., 2018). Likewise, ApoA1-functionalized antioxidant inorganic–organic hybrid nanoplatforms not only reverse local OS but also promote cholesterol efflux, achieving synergistic plaque regression and microenvironment remodeling (Zhang Y. et al., 2025). In nucleic acid-based therapies, cationic liposomes coated with a neutral protective layer and functionalized with VCAM-1-targeting peptides have enabled highly efficient and precise delivery of anti-miR-712. Remarkably, this system achieves effective downregulation of pathogenic gene expression and inhibition of MMP activity at only 20% of the conventional dosage, thereby minimizing off-target toxicity associated with free nucleic acids (Kheirolomoom et al., 2015). Furthermore, integration with photodynamic therapy, which allows spatiotemporally controlled drug release under external light stimulation (Liu et al., 2020), and manganese oxide nanozyme systems loaded with nattokinase (MnOx@NK), which combine thrombolytic and ROS-scavenging functions (Song et al., 2024; Shi et al., 2025), highlights the versatility of nanodelivery platforms. These technologies not only enable safe and localized thrombolysis during the acute phase—substantially reducing the risk of downstream embolic events such as stroke—but also, when combined with injectable hydrogels, effectively reverse adverse ventricular remodeling in the post-MI stage (Xu et al., 2024). Looking ahead, intelligent nanocarriers are poised to play a pivotal role in the comprehensive management of CVDs (Disharoon et al., 2019; Flores et al., 2019; Palazzolo et al., 2020).

#### 4.2.4 Regenerative medicine and cellular engineering

The application of cell-free extracellular vesicles (EVs) in cardiac tissue engineering to promote endogenous myocardial regeneration and therapeutic angiogenesis represents a highly promising frontier in cardiovascular regenerative medicine. The efficacy of conventional cell transplantation is often limited by the hostile microenvironment of infarcted tissue. In contrast, EVs possess the ability to bypass biological barriers and efficiently deliver bioactive molecules to recipient cells, thereby closely recapitulating and even amplifying the therapeutic potential of their parent cells (de Abreu et al., 2020). It has been demonstrated that mesenchymal stem cells derived from human embryonic stem cells exert cardioprotective effects primarily through the secretion of exosomes (50–100 nm) enriched with specific molecular markers. In myocardial ischemia–reperfusion (I/R) injury models, these exosomes significantly reduce infarct size, establishing a paracrine mechanism as the central basis of their therapeutic efficacy (Lai et al., 2010).

Similarly, EVs isolated from induced pluripotent stem cell-derived cardiovascular progenitor cells are enriched with high-abundance microRNAs associated with tissue repair. These EVs have shown superior therapeutic outcomes compared with direct cell transplantation, notably improving left ventricular ejection fraction and reducing ventricular volume in models of congestive heart failure, thereby providing an effective cell-free alternative to conventional cell-based therapies (Liu et al., 2018). In the context of ischemic cardiac revascularization, vesicles secreted by human CD34^+^ cells exhibit intrinsic pro-angiogenic activity, enhancing endothelial cell viability and promoting tubular network formation. These properties position them as critical mediators in progenitor cell-driven therapeutic angiogenesis (Sahoo et al., 2011). However, the rapid clearance of native EVs *in vivo* significantly limits their long-term therapeutic potential. To address this limitation, nanobiomaterials and bioengineering strategies have been introduced to enable targeted accumulation and spatiotemporally controlled delivery. Within tissue engineering approaches, EVs derived from induced pluripotent stem cell (iPS)-derived cardiomyocytes have been incorporated into engineered hydrogel patches. Sustained release of these EVs from the patch applied to infarcted rat hearts not only significantly reduces arrhythmic burden and cardiomyocyte hypertrophy but also promotes sustained recovery of ejection fraction over a 4-week period post-infarction (Wang et al., 2017). To further enhance targeting efficiency, researchers have innovatively fused isolated EVs with platelet membranes via extrusion (P-EVs), thereby conferring inherent inflammation-homing and adhesive properties. In MI/R models, these hybrid vesicles preferentially accumulate in injured vascular endothelial cells, markedly enhancing therapeutic angiogenesis (Li Q. et al., 2021). Additionally, preconditioning parental cells under hypoxic conditions selectively enriches EVs with long non-coding RNA MALAT1. This molecule amplifies cardioprotective effects by targeting miR-497, thereby enhancing cardiomyocyte survival and promoting tubular structure formation in human umbilical vein endothelial cells (Wu et al., 2020).

The integration of highly engineered EVs with advanced nanobiomaterials overcomes fundamental limitations of traditional cell therapies and establishes a promising translational pathway for ischemic heart disease repair. This combinatorial strategy—encompassing targeted surface modification, customizable genetic cargo, and intelligent hydrogel-based delivery—not only significantly reduces myocardial fibrosis but also improves the immunological and vascular microenvironment of the injured myocardium. Despite the substantial regenerative potential demonstrated by bioengineered EVs across various cardiac disease models, several critical challenges remain before full clinical translation can be realized. Future research must address key issues, including standardized large-scale production, long-term storage stability, and precise mechanistic understanding within complex human pathological networks, to facilitate the transition of this “cell-free therapy” from bench to bedside (Zhang et al., 2024; Wu et al., 2026). From the deployment of antioxidant nanozymes to neutralize ROS storms during myocardial ischemia–reperfusion injury, to the use of platelet membrane-mimetic nanoparticles for plaque-specific accumulation of anti-inflammatory agents, nanomedicine has established a comprehensive technological framework spanning early pathological detection to late-stage tissue regeneration.

### 4.3 Limitation

Although this study provides a macroscopic overview of coronary heart disease (CHD) and the field of nanomaterials through systematic bibliometric analysis, several inherent limitations should be acknowledged, which also highlight key challenges for the future development of this research domain. First, potential bias in the search strategy may have resulted in the exclusion of relevant studies, particularly those that did not explicitly adopt standardized nanomaterial-related terminology or were published in non-English-language journals. Second, bibliometric analysis, as a quantitative and descriptive approach, is effective in identifying research hotspots and emerging trends; however, it cannot substitute for in-depth assessments of the scientific rigor or clinical translational potential of individual studies. Third, standard bibliometric indicators should be interpreted with appropriate scientific contextualization. Quantitative clustering based on co-citations and network metrics provides a macro-level structural landscape of the research domain, but cannot directly substitute for granular evaluations of experimental rigor, clinical effect sizes, or intrinsic article quality. Furthermore, raw citation counts are inherently confounded by publication age (temporal window for accumulation), database coverage limits, self-citation practices, and field-specific citation velocities. Crucially, citation-based metrics reflect academic visibility and scholarly discourse within the bibliometric window rather than definitive clinical therapeutic efficacy. Finally, the bibliometric visualization tools employed in this study do not differentiate between author roles (e.g., first author versus corresponding author), nor do they distinguish between different publication types, such as original research articles and review papers produced by highly prolific authors. Instead, authors are ranked primarily based on publication output and co-authorship networks. Consequently, the group of highly productive authors identified herein reflects overall research productivity rather than the specific contributions or individual academic influence of each author.

## 5 Conclusion

This work presents a comprehensive bibliometric and visualization analysis of global research on nanomaterials for CHD therapy from 2012 to 2025. Since 2012, nanotechnology research in CHD has expanded rapidly, accompanied by increasing global participation. China has contributed the largest publication output, while the United States has maintained a prominent position in citation impact and research influence. Nevertheless, international collaboration remains unevenly distributed, highlighting opportunities for broader global cooperation. The evolutionary trajectory reveals a clear shift from early macro-level structural interventions, such as basic tissue engineering and bare-metal scaffolds, to functionalized targeted delivery systems including nanoparticle-mediated drug delivery and extracellular vesicles. Recently, the field has converged around the integrated concept of “pathology-driven precision nanomedicine”. Importantly, recent advances indicate that nanomaterials are increasingly being explored beyond their conventional role as delivery platforms, with growing interest in their potential to modulate the ischemic microenvironment through mechanisms involving ROS regulation, macrophage polarization, and myocardial ischemia-reperfusion injury. These developments highlight an emerging research direction toward mechanism-oriented and disease-specific nanotherapeutic strategies in CHD. Current research frontiers increasingly emphasize translational applications, with biomimetic nanocarriers (e.g., platelet membrane and high-density lipoprotein mimetics), stimuli-responsive hydrogels, and regenerative cell-free approaches representing promising strategies for improving targeting efficiency and therapeutic precision in cardiovascular nanomedicine. However, the transition from laboratory to clinical application requires surmounting traditional material design constraints and addressing key parameters, including large-scale production under Good Manufacturing Practice, long-term *in vivo* safety assessments, and the complex heterogeneity of human atherosclerotic plaques. Consequently, future research should prioritize precision medicine strategies that account for patient-specific pathological differences and explore the integrated application of bioelectronic materials with nanomedicine to reconstruct the electromechanical microenvironment of infarcted myocardium. In conclusion, this study delineates the evolutionary trajectory of nanotherapeutics in CHD. By advocating a holistic approach that integrates advanced materials science with precise pathological insights and clinical needs, this analysis provides a robust scientific foundation for optimizing future research strategies and fostering interdisciplinary collaboration to combat CVDs.

## Data Availability

The original contributions presented in the study are included in the article/Supplementary Material, further inquiries can be directed to the corresponding authors.
