# Supplementary material for: Research trends of nanotechnology in coronary heart disease: a bibliometric analysis from 2012 to 2025
Source: Front Pharmacol. 2026 Sep 30;17:1931509. doi: 10.3389/fphar.2026.1931509 (PMC13662116; doi:10.3389/fphar.2026.1931509)
Supplement: Supplementary file 1 [file Supplementaryfile1.docx]

| **Search Database** | **Search Date** | **Search Topic** | **Search Keywords (TS=Topic Terms)** | **Logical Relationship** | **Search Scope** |
| --- | --- | --- | --- | --- | --- |
| Web of Science Core Collection (WoSCC) | Jan 21, 2026 | Research on the application of nanotechnology in coronary heart disease | **TS** = (("nanoparticle" OR "nanomaterial" OR "nanocarrier" OR "nanomedicine" OR "nanosystem" OR "nanostructure" OR "nanocrystal" OR "nanoformulation") AND ("coronary heart disease" OR "coronary artery disease" OR "ischemic heart disease" OR "myocardial infarction" OR "acute coronary syndrome" OR "coronary disease" OR "CHD" OR "CAD")) | Nanotechnology-related keywords were combined using OR logic, and coronary heart disease-related keywords were also combined using OR logic. The two concept groups were then combined using AND logic. | Database: Web of Science Core Collection (WoSCC); Index: All Editions (including Science Citation Index Expanded [SCI-EXPANDED], Current Chemical Reactions [CCR-EXPANDED], and Index Chemicus [IC]); Publication years: January 1, 2012–December 31, 2025; Document types: Articles and Reviews; Language: English; Excluded: conference abstracts, proceedings papers, editorials, letters, news items, and other non-original publications |
| Scopus | Jan 21, 2026 | Research on the application of nanotechnology in coronary heart disease | **TITLE-ABS-KEY** (( "nanoparticle" OR "nanomaterial" OR "nanocarrier" OR "nanomedicine" OR "nanosystem" OR "nanostructure" OR "nanocrystal" OR "nanoformulation") AND ("coronary heart disease" OR "coronary artery disease" OR "ischemic heart disease" OR "myocardial infarction" OR "acute coronary syndrome" OR "coronary disease" OR "CHD" OR "CAD")) | Same as above | Database: Scopus; Search fields: TITLE-ABS-KEY; Publication years: January 1, 2012–December 31, 2025; Document types: Articles and Reviews; Language: English; Excluded: conference papers, editorials, notes, letters, and other non-original publications. |

Table S1 (Literature Search Strategy)

**Note:** The abbreviations "CAD" and "CHD" were retained in the search strategy because they are widely used abbreviations for coronary artery disease and coronary heart disease in cardiovascular literature. We acknowledge that "CAD" may also refer to computer-aided design in other disciplines. To minimize potential retrieval bias, records in which CAD referred to computer-aided design, CAD/CAM, or other non-cardiovascular applications unrelated to coronary heart disease and nanotechnology were excluded during the screening process.

Supplementary Table S2 (Data Normalization and Standardization Procedures)

magnetic nanoparticle	magnetic nanoparticles

nanoparticle	nanoparticles

gold nanoparticle	gold nanoparticles

nanocarrier	nanocarriers

nanoparticle mediated delivery	nanoparticle-based delivery

drug delivery system	drug delivery systems

silica nanoparticle	silica nanoparticles

superparamagnetic iron oxide	superparamagnetic iron oxide nanoparticles

lipid nanoparticle	lipid nanoparticles

polymeric nanoparticle	polymeric nanoparticles

silica nanoparticle	silica nanoparticles

composite nanoparticle	composite nanoparticles

mesoporous silica nanoparticle	mesoporous silica nanoparticles

gold nanorod	gold nanorods

nanostructure	nanostructures

biomarker	biomarkers

heart failure	heart failure disease

myocardial infarction	heart attack

cardiac regeneration	heart regeneration

cardiomyocyte	cardiac myocyte

stem cell	stem cells

mesenchymal stem cell	MSC

extracellular vesicle	extracellular vesicles

endothelial cell	endothelial cells

vascular progenitor cell	vascular progenitor cells

dendritic cell	dendritic cells

macrophage macrophages

fibroblast	fibroblasts

apoptosis	apoptosis	cell

biomaterial	biomaterials

oxidative stress	oxidative stresses

targeted therapy	targeted therapies

gene therapy	gene therapies

therapeutic delivery	therapy delivery

nanoparticle therapy	nanoparticle-based therapy

drug eluting stent	drug-eluting stent

hydrogel	hydrogels

polymer-based nanoparticle	polymer-based nanoparticles

3D printing	three-dimensional printing

coronary heart disease	coronary artery disease

cardiovascular disease	cardiovascular diseases

cardiovascular disease	cardiovascular disease (cvd)

cardiovascular disease	cardiovascular diseases (cvds)

myocardial infarction	acute myocardial infarction

ischemia-reperfusion injury	ischemia/reperfusion injury

ischemia-reperfusion injury	ischemic/reperfusion injury

ischemia-reperfusion injury	ischemia reperfusion injury

nanoparticle	nanoparticles

stem cell	stem cells

endothelial cell	endothelial cells

endothelial progenitor cell	endothelial progenitor cells

biomarker	biomarkers

c reactive protein	c-reactive protein

inflammation	inflammatory response

detection	sensitive detection

detection	ultrasensitive detection

biosensor	immunosensor

biosensor	electrochemical biosensor

magnetic resonance	magnetic resonance imaging

extracellular vesicle	extracellular vesicles

extracellular vesicle	extracellular vesicles exosm

gene therapy	gene transfer

sirna	small interfering rna

scaffold	nanofibrous scaffold

magnetic nanoparticle	magnetic nanoparticles

UNITED STATES	usa

UNITED KINGDOM	wales

UNITED KINGDOM	north Ireland

UNITED KINGDOM	england

UNITED KINGDOM	scotland

CHINA	taiwan

CHINA	hongkong

CHINA	peoples r china

TURKIYE	TURKEY

U ARAB EMIRATES	UNITED ARAB EMIRATES

Brigham & Womens Hosp	Brigham & Womens Hosp Dept Cardiovasc Med

China Med Univ	China Med Univ & Hosp

Chinese Acad Med Sci	Peking Union Med Coll

Natl Univ Singapore	Yong Loo Lin Sch Med

Cardioctr Ticino Fdn	Cardioctr Ticino

**Supplementary Figure**

**Fig. S1** Network visualization of keywords with a frequency of no less than 20 times.
